# Distinct Binding and Immunogenic Properties of the Gonococcal Homologue of Meningococcal Factor H Binding Protein

**DOI:** 10.1371/journal.ppat.1003528

**Published:** 2013-08-01

**Authors:** Ilse Jongerius, Hayley Lavender, Lionel Tan, Nicola Ruivo, Rachel M. Exley, Joseph J. E. Caesar, Susan M. Lea, Steven Johnson, Christoph M. Tang

**Affiliations:** 1 Sir William Dunn School of Pathology, University of Oxford, Oxford, United Kingdom; 2 Centre for Molecular Microbiology and Infection, Imperial College, London, United Kingdom; Faculté de Médecine Paris Descartes, site Necker, France

## Abstract

*Neisseria meningitidis* is a leading cause of sepsis and meningitis. The bacterium recruits factor H (fH), a negative regulator of the complement system, to its surface via fH binding protein (fHbp), providing a mechanism to avoid complement-mediated killing. fHbp is an important antigen that elicits protective immunity against the meningococcus and has been divided into three different variant groups, V1, V2 and V3, or families A and B. However, immunisation with fHbp V1 does not result in cross-protection against V2 and V3 and *vice versa*. Furthermore, high affinity binding of fH could impair immune responses against fHbp. Here, we investigate a homologue of fHbp in *Neisseria gonorrhoeae*, designated as Gonococcal homologue of fHbp (Ghfp) which we show is a promising vaccine candidate for *N. meningitidis*. We demonstrate that Gfhp is not expressed on the surface of the gonococcus and, despite its high level of identity with fHbp, does not bind fH. Substitution of only two amino acids in Ghfp is sufficient to confer fH binding, while the corresponding residues in V3 fHbp are essential for high affinity fH binding. Furthermore, immune responses against Ghfp recognise V1, V2 and V3 fHbps expressed by a range of clinical isolates, and have serum bactericidal activity against *N. meningitidis* expressing fHbps from all variant groups.

## Author Summary

*Neisseria meningitidis* is a major cause of sepsis and meningitis in young children and adolescents. Although vaccines are currently available against several serogroups, a broadly effective vaccine against serogroup B is still needed. Factor H binding protein (fHbp) can bind the human complement regulator factor H (fH) and is an important meningococcal immunogen. fHbp is divided into three variant groups (V1, V2 and V3) and immunisation with V1 fHbp does not elicit cross-protection against meningococcus expressing fHbp V2 or V3, and *vice versa*. Here, we investigate a homologue of fHbp in *Neisseria gonorrhoeae* which we named Gonococcal homologue of factor H binding protein (Ghfp). We show that in contrast to fHbp, Ghfp is not expressed on the bacterial surface and is unable to bind to factor H. Surprisingly, we found that antibodies raised against Ghfp have the capacity to mediate protective immunity against *N. meningitidis* expressing any of the three variant groups of fHbp, and could provide a broadly protective vaccine against *N. meningitidis*.

## Introduction

The Gram negative bacterium *Neisseria meningitidis* is part of the normal human nasopharyngeal flora in up to 40% of healthy individuals [1], [2] and a leading cause of sepsis and meningitis worldwide, with a case fatality rate from septicaemia of approximately 10% [3], [4]. Because of the non-specific early symptoms and rapid progression of meningococcal disease, there is an urgent need to develop vaccines to protect individuals from this important infection [4], [5]. *N. meningitidis* is classified into 12 different serogroups based on its polysaccharide capsule, although only six serogroups are responsible for the majority of disease. Currently there are vaccines based on the polysaccharide capsule of four of these serogroups (*i.e.* A, C, W, and Y) [5]. However, the capsule of serogroup B *N. meningitidis* (MenB) is structurally identical to a modification of a cell adhesion molecule present in the foetal brain, and is thus weakly immunogenic and could induce autoimmunity if used as a vaccine [6]. Vaccines based on outer membrane vesicles have proven to be effective against MenB but only in combating epidemic disease caused by a single clone [7]; the most effective approach to produce a broadly protective vaccine against all *N. meningitidis* serogroups (including MenB) will be the use of protein based vaccines [8].

Factor H binding protein (fHbp) of *N. meningitidis* is an important component of MenB vaccines currently under advanced clinical development [8], [9]. Immunisation with fHbp elicits serum bactericidal antibodies [8], [9], a marker of protection, and the protein provides an important mechanism for immune evasion for the meningococcus by recruiting the negative complement regulator, factor H (fH), thereby protecting *N. meningitidis* against complement-mediated lysis [10], [11]. fHbp is a surface expressed lipoprotein consisting of two *β* barrels [12], [13]. Based on sequence alignments, fHbp has been categorised into three different variant groups, V1, V2 and V3, or two families, A and B [8], [9]. However, immunisation with V1 fHbp (family B) does not elicit bactericidal responses against V2 and V3 (family A) fHbp-expressing strains and *vice versa* [8], [9], [14]. In addition, immunisation with one V1 peptide does not provide cross-protection against all strains expressing V1 fHbps [14]. This suggests that a broadly protective vaccine should include multiple fHbps, or fHbps which elicit cross-protection. Current vaccines contain V1.1 fHbp together with other antigens, or a combination of V1 and V3 fHbps [8], [9].

Although *Neisseria gonorrhoeae* binds fH to its surface, the receptor on the bacterium is Por1A which is not related to fHbp [15]. However, inspection of gonococcal genome reveals a homologue of *fhbp* (annotated as *ngo0033* in *N. gonorrhoeae* strain FA1090); we designated the predicted protein Gonococcal homologue of fHbp (Ghfp), because it is approximately 90% identical to V3 fHbps. In contrast to meningococcal fHbp, Ghfp is highly conserved with three alleles described which only differ by one or two amino acids [16]. Furthermore, Ghfp is not predicted to contain a signal sequence or a lipid modification motif (LXXC) suggesting it is unlikely to be expressed on the bacterial surface [16], [17]. Here, we investigate the location, the fH binding capacity and the vaccine potential of Ghfp.

## Results

### Ghfp is not surface expressed

**Figure 1:**
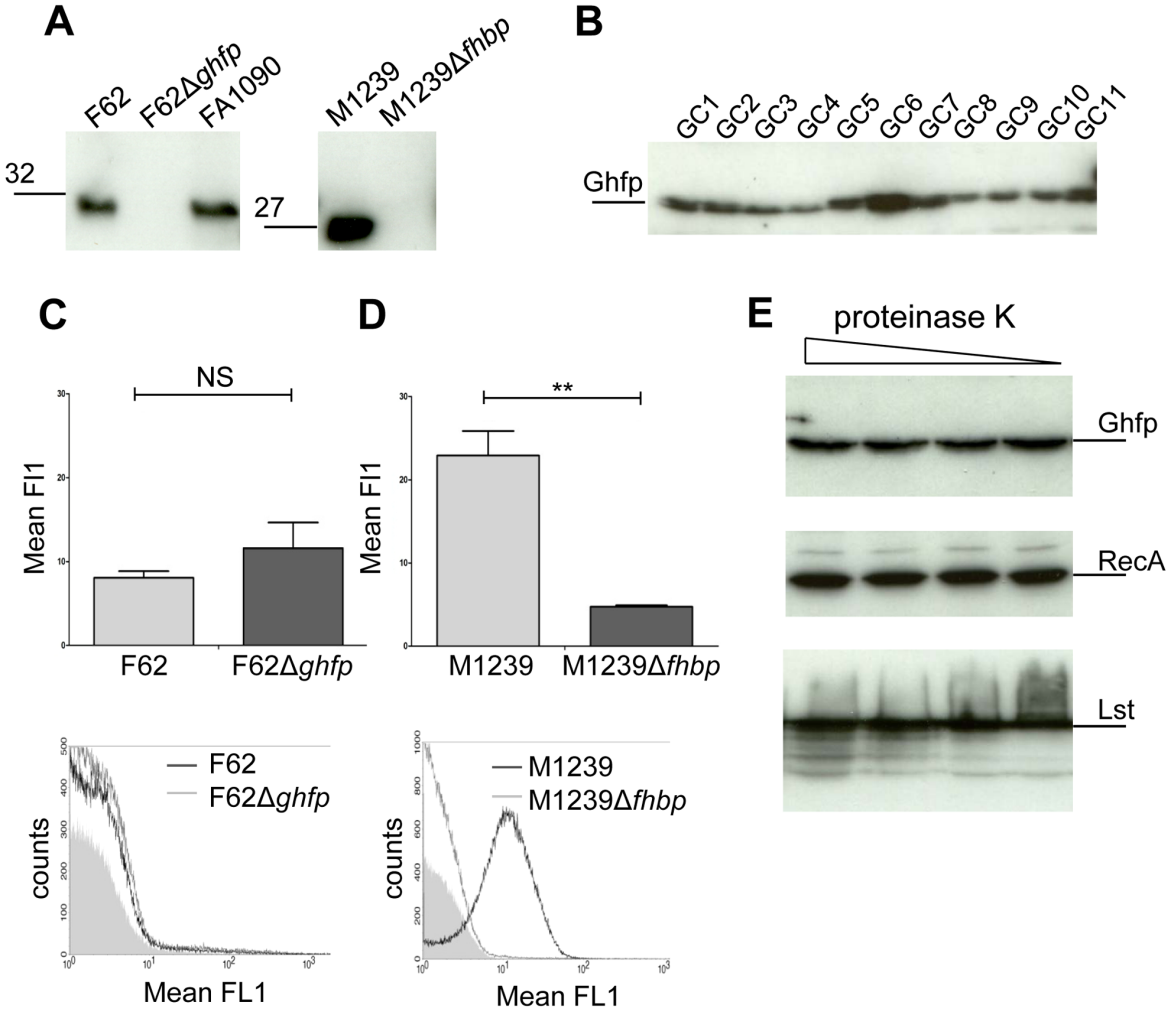
Ghfp is not expressed on the surface of *N. gonorrhoeae*. (**A**) Western blot analysis of Ghfp expression by *N. gonorrhoeae* strains F62, F62Δ*ghfp* and FA1090, and fHbp V3.28 expressed by *N. meningitidis* strains M1239 and M1239Δ*fhbp* using anti-Ghfp serum. (**B**) Western blot analysis of Ghfp expressed by a panel of clinical *N. gonorrhoeae* isolates (GC1-11, inclusive). Surface expression of Ghfp (**C**) and fHbp V3.28 (**D**) was assessed by flow cytometry analysis using anti-Ghfp serum. Error bars, SEM of three separate experiments; ** p<0.05 and NS (not significant, Student's *t*-test). Representative flow cytometry overlays are shown below the graphs. Bacteria incubated without anti-Ghfp serum are shown as the grey filled areas. (**E**) Bacteria were exposed to proteinase K (3 ng/ml and serial three-fold dilutions) and the effect on proteins (shown) determined by Western blot analysis using anti-Ghfp, anti-RecA and anti- α-Lst serum.

Analysis of the genome sequence of *N. gonorrhoeae* strain FA1090 identified the presence of a *fhbp* homologue [16], [17] which we designated Gonococcal homologue of fHbp , Ghfp. Sequence alignment of Ghfp with available fHbp sequences (www.neisseria.org) reveals that Ghfp has between 60–67%, 81–89% and 86–94% amino acid identity with V1, V2 and V3 fHbps, respectively (Supplementary Figure S1). To investigate the cellular location of Ghfp, sera were raised against recombinant Ghfp from *N. gonorrhoeae* strain FA1090. By Western blot analysis, sera recognised a protein with an estimated molecular weight of 30 kDa (corresponding to Ghfp) in lysates of *N. gonorrhoeae* strains FA1090 and F62; no protein was detected in lysates from F62Δ*ghfp* (Figure 1A). Sera raised against Ghfp also recognise V3.28 fHbp expressed by *N. meningitidis* strain M1239 (Figure 1A). Moreover, Ghfp was expressed by 20 clinical *N. gonorrhoeae* strains isolated in the UK (Figure 1B and not shown). To determine whether Ghfp is surface located, we performed flow cytometry analysis with anti-Ghfp serum to detect Ghfp on the surface of *N. gonorrhoeae* F62 and F62Δ*ghfp*, and fHbp on *N. meningitidis* M1239 and M1239Δ*fhbp* (Figure 1C and 1D). Results demonstrate that anti-Ghfp serum recognises V3.28 fHbp on the surface of *N. meningitidis*, but there was no detectable Ghfp on the gonococcal surface.

To exclude the possibility that the lack of detection of Ghfp by flow cytometry was due to low expression levels, we also exposed viable bacteria to proteinase K, and monitored the degradation of Ghfp, a surface protein *i.e.* the α-2,3-sialyltransferase, Lst [18], and the cytoplasmic protein RecA by Western blot analysis. Ghfp and RecA were unaffected by exposing cells to proteinase K. The relative amounts of full length protein after incubation with reducing concentrations of proteinase K (serial three-fold dilutions from 3 ng/ml, Figure 1E) were: Ghfp, 94-90-89-100; RecA, 101-98-101-100. In contrast, digestion of Lst was observed : the amount of Lst peptides with reducing concentrations of proteinase K were 214-158-128-100 (Figure 1E). Recombinant Ghfp is cleaved by these concentrations of proteinase K (data not shown) demonstrating that Ghfp is susceptible to cleavage by this protease. In conclusion, our results demonstrate that Ghfp is expressed by *N. gonorrhoeae* but is not located on the bacterial surface, in keeping with previous predictions.

### Identification of residues responsible for low affinity binding of Ghfp to fH

**Figure 2:**
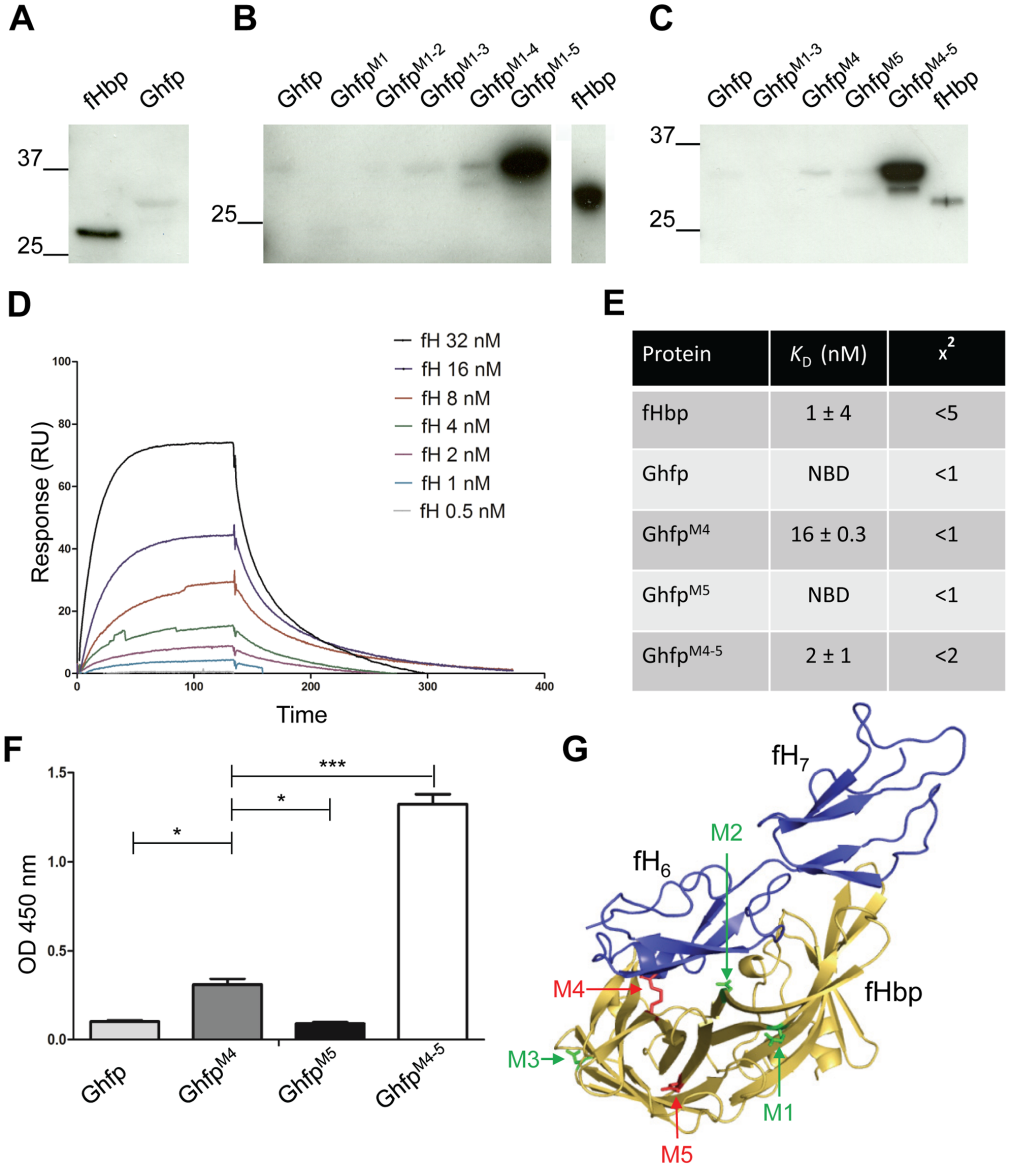
fH binding capacity of Ghfp. (**A-C**) fH binding to wild type and modified Ghfp and fHbp V3.45 was assessed by far Western analysis using normal human serum as the source of fH. Western blots are representatives of three separate experiments. Molecular mass is shown in kDa. (**D**) Typical equilibrium fit for binding of fH_6–7_ to Ghfp^M4–5^. (**E**) SPR was performed with Ghfp, Ghfp^M4^ (R288H), Ghfp^M5^ (D318G) and Ghfp^M4–5^ (R288H/D318G); NBD, no binding detected. (**F**) Detection of full length fH (5 nM) binding to wild-type and modified Ghfp by ELISA. Error bars, SEM of three separate experiments; *** p<0.01 and * p<0.1 (Student's *t*-test). (**G**) Cartoon presentation of the predicted structure of Ghfp (Yellow) and fH (Blue). Residues M1 (R176), M2 (D199) and M3 (D212) are shown in green while those amino acids that are important for fH binding, M4 (R288) and M5 (D318G), are shown in red.

Due to its high sequence identity with V3 fHbp, which binds fH with a *K*_D_ in the nM range [13], [14], fH binding to Ghfp was tested by far Western analysis. Surprisingly, there was no detectable fH binding to Ghfp using normal human serum as the source of fH (Figure 2A). Therefore, we compared the sequence of Ghfp with V2 and V3 fHbps that bind fH at high affinity, and identified five amino acids that are consistently different between Ghfp and V2/V3 fHbps *i.e.* R176, D199, D212, R288 and D318 of Ghfp (amino acid numbering according to fHbp V1.1 structure [12], Supplementary data Figure S2). Recent studies have shown that Ghfp is highly conserved [16] which we confirmed by sequencing Ghfp in a panel of 20 clinical isolates from the UK (not shown). All three Ghfp polymorphisms in our isolates had been identified previously [16] and are also present in V2 and/or V3 fHbp, so do not include residues (R176, D199, D212, R288 and D318) that are unique to Ghfp. The amino acids R176 and D199 are located in the predicted N-terminal barrel of Ghfp and, similar to C-terminal *β* barrel residue D212, are not located at the region of Ghfp corresponding to the interface of fHbp with fH [13]. In contrast, R288 is located in close proximity to the predicted fH:Ghfp interface, while D318 could be involved in interactions between the two predicted *β* barrels of Ghfp. To determine whether these five amino acid changes are responsible for the reduced fH binding to Ghfp, we modified these specific amino acids into the equivalent residues in the closely related V3.45 fHbp. Modification of all five residues in Ghfp^M1–5^ (*i.e.* R176Q, D199G, D212S, R288H and D318G) was sufficient to enable Ghfp to bind fH by far Western analysis (Figure 2B). Analysis of Ghfp^M1^ (R176Q), Ghfp^M1–2^ (R176Q and D199G), Ghfp^M1–3^ (R176Q, D199G and D212S) and Ghfp^M1–4^ (R176Q, D199G, D212S and R288H) demonstrated that these modifications did not restore fH binding. However the substitutions R288H and D318G in Ghfp^M4–5^ are sufficient to confer fH binding to Ghfp by far Western analysis (Figure 2C). To further analyse this interaction in more detail, the binding of Ghfp and V3.45 fHbp to fH_6–7_ was also investigated by Surface Plasmon Resonance (SPR, Figures 2D and 2E). The dissociation constant of V3.45 fHbp and complement control protein (CCP) domains 6 and 7 of fH (fH_6–7_) was 1±4 nM, similar to previous results for V3 fHbps [13], [14]. Consistent with our far Western analysis, no fH binding was detected to Ghfp by SPR under these conditions. Moreover, no fH binding was observed to Ghfp^M5^ (D318G). There was fH binding detected to Ghfp^M4^ (R288H) (*K*_D_ of 16±0.3 nM) while the double substitution, Ghfp^M4–5^ (R288H and D318G), resulted in fH binding that was equivalent to fHbp (*K*_D_ *i.e.* of 2 nM).

To exclude the possibility that Ghfp interacts with fH via CCP domains other than fH_6–7_, we also examined fH binding by ELISA in which recombinant proteins were coated on the wells of plates and binding to purified full length fH was detected. Consistent with SPR, we observed fH binding to Ghfp^M4–5^, partial fH binding to Ghfp^M4^, and no fH binding to wild-type Ghfp or Ghfp^M5^ (Figures 2E). In conclusion, despite its high amino acid identity with V3 fHbp, Ghfp does not bind fH to any significant degree, and there are only two amino acids responsible for the striking difference in affinity compared with fHbp. Due to its high sequence identity with V3 fHbp, we were able to map the Ghfp sequence on our V3 fHbp structure [13]. Figure 2G shows the location of these two important amino acids, R288 (altered in M4) and D318 (M5); while R288 lies on the face of Ghfp which interacts with fH in fHbps, D318 may influence the interaction between the two *β* barrels of the protein.

### fH binding to modified V3.45 fHbp

**Figure 3:**
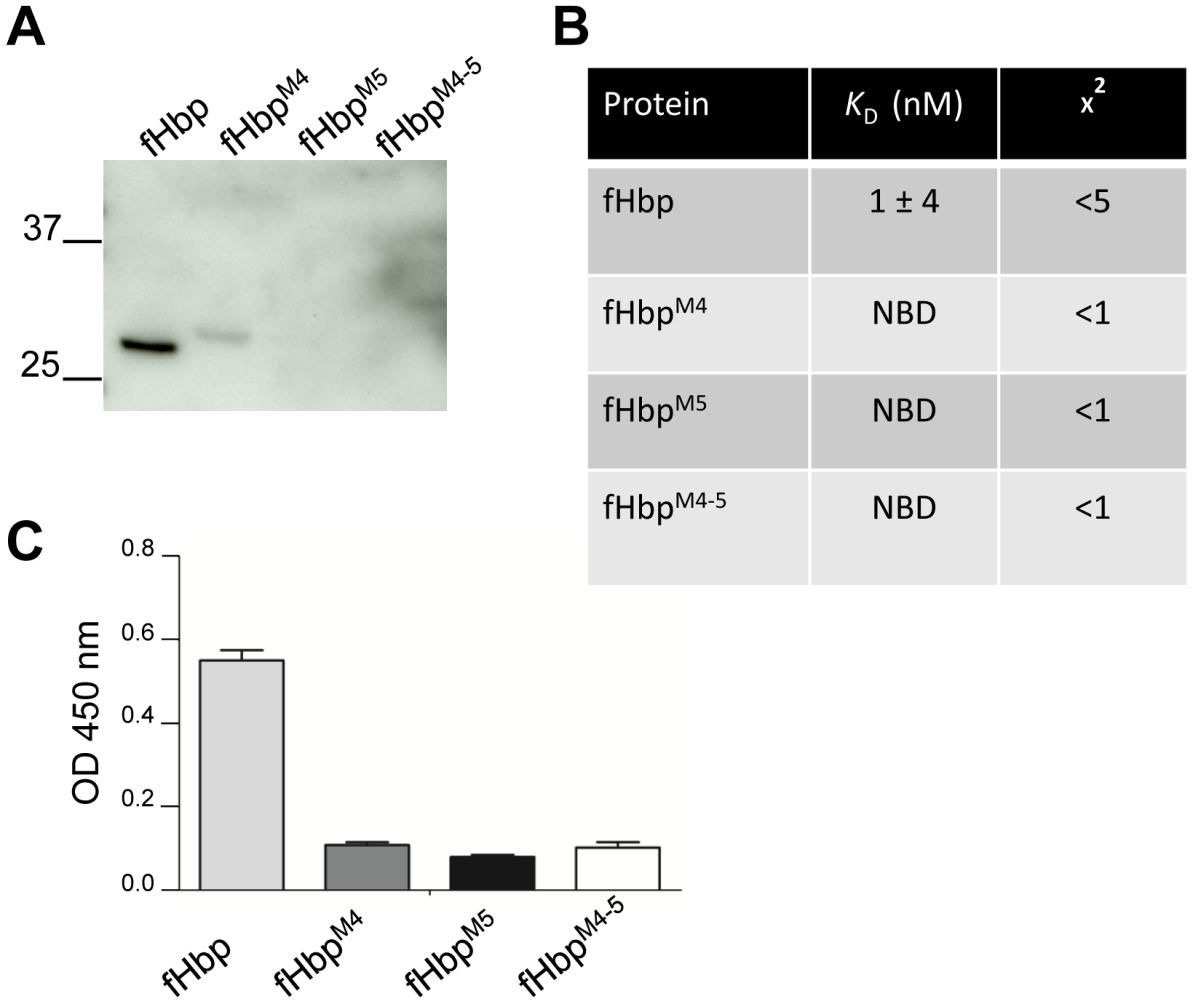
fH binding to modified fHbp V3.45. (**A**) Analysis of fH binding to modified V3.45 fHbp by far Western using normal human serum as the source of fH. Molecular mass is shown in kDa. (B) SPR values of fH_6–7_ binding to wild type and modified V3.45 fHbp; NBD, no binding detected. (**C**) Detection of full length fH (5 nM) binding to wild-type and modified V3.45 fHbp by ELISA. Data represents the mean ± SEM of three different experiments.

As the modifications R288H and D318G in Ghfp are sufficient to confer high affinity fH binding, we next investigated whether the corresponding residues in V3.45 fHbp are necessary for binding to fH. We generated V3.45 fHbp with H288R and G318D (fHbp^M4–5^); these modifications were not included in our recent analysis of fH:V3 fHbp interactions which involved alanine substitution of fHbp [13]. Initially binding of fH was examined by far Western analysis (Figure 3A) and showed loss of detectable fH binding to fHbp^M4^ (H288R), fHbp^M5^ (G318D) or fHbp^M4–5^ (H288R and G318D). To verify these results, binding of fH_6–7_ to fHbp wild type and modified proteins was analysed by SPR (Figure 3B). No detectable binding of fH was observed to fHbp^M4^, fHbp^M5^ and fHbp^M4–5^ under these conditions, demonstrating that both of these residues are necessary for high affinity interactions with fH. To exclude the possibility that these modified fHbp molecules interact with fH via CCP domains other than fH_6–7_, we examined binding to purified full length fH by ELISA. Consistent with SPR, we observed fH binding to fHbp V3.45 but no binding to fHbp^M4^, fHbp^M5^ and fHbp^M4–5^ (Figure 3C). Taken together, we conclude that the amino acids present at positions 288 and 318 are the basis for the profound difference in interactions with fH observed in the closely related proteins from the gonococcus and meningococcus.

### Immunogenicity of Ghfp

**Figure 4:**
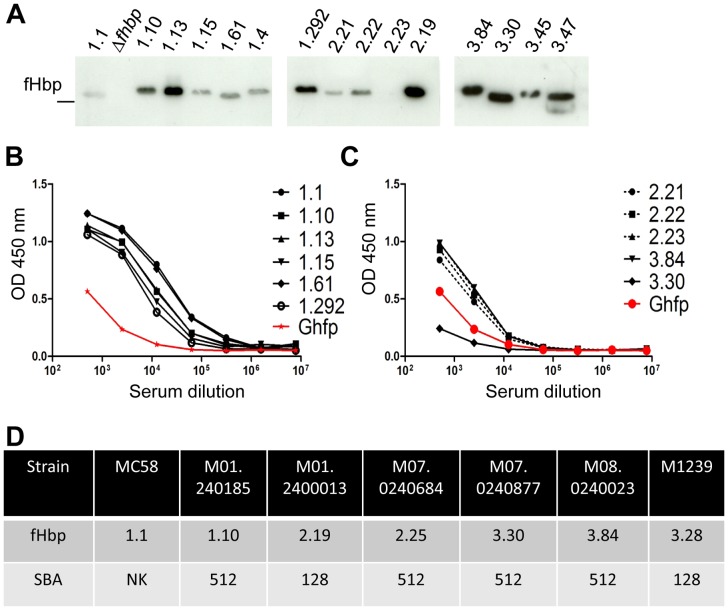
Immunogenicity of Ghfp. (**A**) Detection of fHbp variants in whole cell lysates of *N. meningitidis* by Western blot analysis using anti-Ghfp serum. Recognition of recombinant V1 (**B**), and V2and V3 (**C**) fHbps by anti-Ghfp serum by ELISA. (**D**) SBA responses for anti-Ghfp serum.

Next we investigated the vaccine potential of Ghfp by examining the ability of sera raised against this protein to recognise fHbps expressed by a range of *N. meningitidis* isolates. Immune sera not only recognise closely related V3 fHbps expressed in whole cell extracts of *N. meningitidis* but also V1 and V2 proteins (Figure 4A). However, V2.23 fHbp expressed by *N. meningitidis* strain 5/99 was not detected by anti-Ghfp serum or by sera raised against V2 fHbp [19], suggesting that this strain expresses little or no fHbp. Therefore, we examined whether anti-Ghfp serum recognises equivalent amounts of different recombinant fHbps by ELISA. Surprisingly, immune sera raised against Ghfp detected all V1, V2 and V3 fHbps examined (Figure 4B and C). Serum bactericidal activity (SBA) is an established correlate of protective immunity against serogroup C meningococcal infection [8]. To determine whether immunisation with Ghfp elicits functional immune responses, we evaluated the SBA of anti-Ghfp serum against several *N. meningitidis* strains expressing V1, V2 and V3 fHbp (Figure 4D). We found SBA against strains expressing V1, V2 and V3 fHbps.

**Figure 5:**
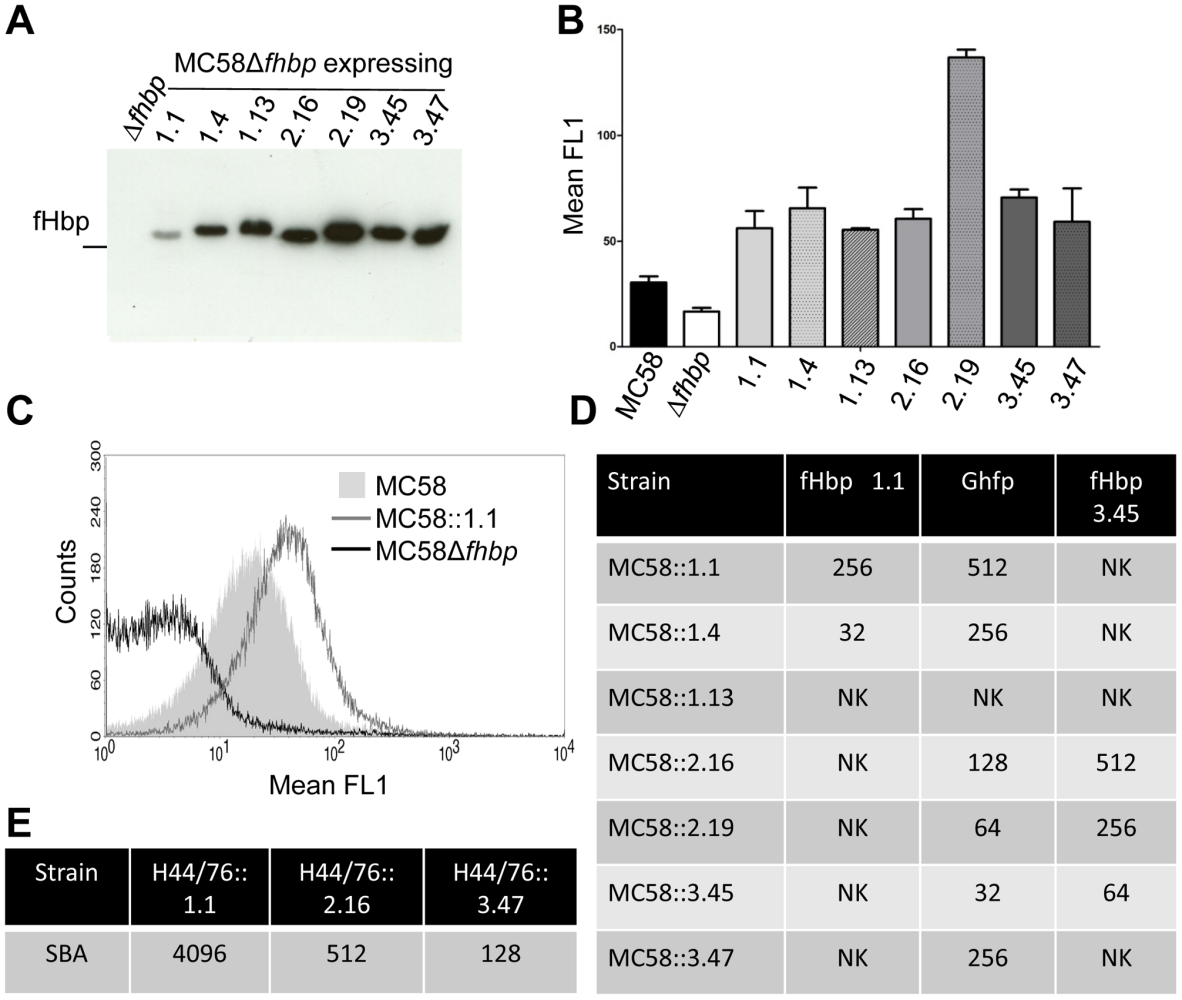
Immune responses against isogenic *N. meningitidis* strains. (**A**) Western blot analysis of whole cell lysates of fHbp expressed by isogenic MC58Δ*fhbp* strains detected by anti-Ghfp serum. (**B**) Surface expression of different fHbps in the isogenic MC58Δ*fhbp* strains detected by flow cytometry. Graph shows the mean ± SEM of three separate experiments. (**C**) Representative corresponding flow cytometry overlay of MC58 (grey hatched area), MC58Δ*fhbp* and MC58Δ*fhbp*+*fhbp* V1.1 detected by anti-Ghfp by flow cytometry. (**D**) SBA responses against isogenic MC58 strains for anti-Ghfp, fHbp V1.1, and fHbp V3.45 serum using rabbit complement; NK, no killing. (**E**) SBA responses against isogenic H44/76 strains with anti-Ghfp serum using rabbit complement.

SBA can be influenced by expression levels of fHbp or inherent serum resistance of the bacteria due to capsule expression [20]. To study the observed cross-protection of anti-Ghfp serum independent of these factors, we constructed isogenic strains of *N. meningitidis* MC58 each expressing one of the seven most prevalent fHbps (*i.e.* V1.1, V1.4, V1.13, V2.16, V2.19, V3.45 and V3.47) from disease isolates in England and Wales, accounting for 70% of cases [19]. To this end, we inactivated the wild-type copy of *fHbp* and introduced a single copy of the gene encoding each of the selected variants at an ectopic site under the control of an IPTG inducible promoter. Expression of the different fHbps was confirmed by Western blot analysis of whole cell extracts (Figure 5A) and surface expression verified by flow cytometry (Figure 5B and C), showing higher expression levels compared to wild type fHbp expression. We determined the SBA of anti-Ghfp serum against the isogenic *N. meningitidis* strains and compared the findings with sera raised against V1.1 or V3.45 fHbp (Figure 5D). We found that anti-Ghfp serum exhibited SBA against *N. meningitidis* expressing V1.1, V1.4, V2.16, V2.19, V3.45 and V3.47. In contrast, anti-fHbp V1.1 serum only elicited SBA responses against V1.1 and V1.4 expressing strains, while anti-V3.45 fHbp serum had SBA against all V2 as well as the V3.45 expressing strains (*i.e.* strains expressing family A proteins). No detectable SBA was measured with any sera against the isogenic MC58Δ*fhbp* strain or using sera from mice receiving adjuvant alone (data not shown). To examine this cross-protection in another genetic background, we constructed isogenic strains of *N. meningitidis* H44/76 in a similar way to express V1.1, V2.16 or V3.47, and observed similar cross-protective SBA responses (Figure 5E). In conclusion, Ghfp can elicit SBA against V1, V2 and V3 fHbp expressing *N. meningitidis* and is therefore a naturally occurring protein capable of providing cross-protection.

## Discussion

*N. meningitidis* and *N. gonorrhoeae* are two human specific, closely related pathogens that inhabit distinct niches in the body. *N. gonorrhoeae* causes sexually transmitted infections predominantly affecting the mucous membranes of the genito-urinary tract, while *N. meningitidis* colonises the nasopharynx [21]. Despite sharing many similarities of the genetic level, these bacteria employ entirely different mechanisms to evade immune responses, and in particular, to avoid complement activation on their surface [22]. For example, disease isolates of *N. meningitidis* express a polysaccharide capsule which is essential for high-level serum resistance [23], while *N. gonorrhoeae* is not encapsulated. Instead sialylation of lipopolysaccharide markedly promotes complement resistance in the gonococcus [24] but this has less impact on *N. meningitidis* [25].

Both organisms have evolved to bind fH to their surface to prevent complement activation (by down-regulating the alternative pathway) but use different strategies. The gonococcus recruits fH via an exposed surface loop of Por1A (loop 5), an outer membrane porin often expressed by isolates recovered from patients [15]. fH can also bind to gonococci expressing Por1B albeit to a lesser degree, with this interaction facilitated by lipopolysaccharide sialylation [26]. Although meningococci express class 3 and class 2 porins (which are related to Por1A and Por1B of *N. gonorrhoeae*, respectively), these are not involved in fH binding; loop 5 of the meningococcal porins lacks a region present in gonococcal Por1A, which probably accounts for its inability to bind fH [27]. Instead, the surface expressed lipoprotein fHbp mediates high affinity binding of fH by the meningococcus irrespective of variant group [13]. This interaction enhances bacterial survival in whole blood and prevents serum dependent killing [10], [11]. It is not clear why the organisms have adopted alternative approaches to exploit the same human molecule, but it is likely to be influenced by the affinity of the interaction, the local availability of fH and the density of the bacterial receptor, as well as other factors conferring complement resistance. Without capsules, gonococci are largely reliant on their capacity to recruit fH and C4bp to survive in the human host [28], [29]. Therefore the relatively low levels of fH in the genito-urinary tract may have favoured its recruitment by a highly abundant protein on the gonococcal surface, such as porin.

Here, we show that Ghfp, the gonococcal homologue of the meningococcal fH receptor, does not bind fH to any detectable extent despite its high sequence identity with fHbp. Remarkably, only two amino acids in Ghfp (R288 and D318) that differ from those in fHbp are responsible for this lack of interaction. Furthermore, the replacement of the equivalent amino acids in V3.45 fHbp (*i.e.* H288R and G318D) resulted in loss of fH binding. The H288R modification is located at the fH:fHbp interface; the side chain of fHbp H288 sits in a hydrophobic pocket in fH formed by H337, Y353 and the methylene groups of the R341. The extended side chain of Ghfp R288 is too long to fit into this pocket without remodelling the interface, and would also result in electrostatic repulsion with R341 of fH. The lack of fH binding to V3.45 fHbp^M5^ (*i.e.* G318D) is more difficult to explain as it is located away from the fH:fHbp interface, and is at the end of the final strand of the second *β* barrel. However, the register of this strand is such that the side chain of residue 318 points into the hydrophobic core of the barrel. Substitution of Gly with Asp is not possible without structural rearrangement due to steric clashes in the hydrophobic core as it is energetically unfavourable to place a negative charge in the hydrophobic environment. Given this final strand also makes crucial contacts with the first *β* barrel, this substitution could lead to structural rearrangements at interface between the two barrels and therefore alter the distal fH binding site (which comprises both barrels). Recently, we identified several residues in V1, V2 and V3 fHbps which are needed for high affinity interactions with fH through alanine scanning mutagenesis [13]. Here we found two further mutations that abolish fH:fHbp binding by analysing the binding characteristics of a natural protein.

While fHbp is located on the surface of the meningococcus, we demonstrate that Ghfp is not on the external surface of the gonococcus, as suggested previously [17]. Examination of Ghfp also reveals the absence of a signal sequence for export so the protein is likely to remain intracellular and not secreted into the extracellular milieu. We can however not exclude the possibility that the location of Ghfp changes during infection. The function of Ghfp remains unknown, although due to its high level of identity to fHbp, we cannot exclude that Ghfp promotes survival in the presence of antimicrobial peptide LL-37 [30] or has a role in siderophore binding [31]. However, these are unlikely functions for Ghfp given the location of the protein.

fHbp is a key component of protein sub-unit meningococcal vaccines under late phase clinical development [8], [9], [32]–[34]. Unfortunately, antibody responses against fHbp are thought to be largely variant/family specific. Therefore fHbp-based vaccines consisting of a single natural fHbp might be expected to have limited coverage. To overcome this issue, vaccines under development have included fHbp together with other antigens namely GNA2132, NadA, GNA1030, GNA2091 and a membrane vesicle [35], or multiple fHbp variants [9]. Here, we show that anti-sera raised against Ghfp has the potential to recognize representative V1, V2 and V3 fHbps, in contrast to sera raised against the widely used V1.1 fHbp and V3.45 fHbp [8]. More importantly, we showed that Ghfp has the potential to elicit SBA against wild-type *N. meningitidis*, and two different strains expressing the most common V1, V2 and V3 fHbps. SBA of murine immune sera was assayed in the presence of rabbit complement. Although a heterologous non-human source, rabbit complement has been used to validate the immunogenicity of conjugate vaccines in pre-clinical and clinical studies [36]. Furthermore, V1 fHbps differ in their recognition by sera raised against Ghfp (Figure 4A) but this cannot be explained at the level of overall sequence identity as the proteins we examined are all approximately 60% identical to Ghfp. The advantage of using isogenic strains over naturally occurring isolates is that this approach allows analysis of the effect of diversity in protein sequence on cross-protective responses, while excluding strain-specific, confounding factors such as levels of expression and other mechanisms of immune escape [37]. This breadth of activity was an unexpected finding which has been seen with synthetic fHbp molecules containing epitopes from different fHbp variants [38], [39] and is not the result of overexpression of fHbp in our isogenic strains; anti-sera raised against V1.1 only has SBA against V1.1 and V1.4 in line with previous results [14], while our work showed that sera raised against V3.45 was able to elicit SBA against strains expressing V2.16 and V2.19, again consistent with previous work demonstrating that raised anti-V3.45 fHbp serum does have SBA against some V2 fHbp expressing strains [14].

The mechanism underlying the broad protection of Ghfp is currently unknown. Based on the position of invariant amino acids in different fHbps, the protein has also been divided into five variable segments, designated as V_A_-V_E_. Using these five segments, fHbps can be categorized into six modular groups [40]. A closer inspection of the fHbps expressed in our isogenic strains reveals that all alleles and Ghfp harbour an identical variable segment D (V_D_). However, this common sequence cannot be the basis of the cross-protection offered by Ghfp as fHbp V3.45 and V1.1 also harbour this region yet do not provide the same breadth of SBA. Another possibility is that the immunogenic properties of Ghfp are not solely dependent on its primary amino acid sequence but instead a result from its conformation. For instance, it is possible that due to D318 or other residues, the folding of Ghfp is altered compared with V3.45, altering accessibility of certain regions of fHbp to B cell receptors and therefore inducing cross-protective responses. We also compared Ghfp and fHbp V3.45 with the rationally designed fHbp that showed broad cross-protection due to introduction of V2 and V3 epitopes into V1.1 fHbp [39]. However all the amino acids introduced into V1.1 to obtain the cross-protection are present in both Ghfp and fHbp V3.45 and so cannot explain the cross-protection we observed with Ghfp.

In summary, Ghfp is a promising vaccine candidate against *N. meningitidis* since the protein not only offers a broad range of protection, but is also a naturally occurring non-fH binding molecule. There are potential drawbacks for the use of functional fHbps as a vaccine antigen due to its high affinity binding with fH. The extensive binding of fH to fHbp could shield immunogenic epitopes on the antigen resulting in less effective antibody responses [12]. Moreover, binding of fHbp to fH might reduce the immunogenicity at the site where antibody responses are initiated [41] or it could lead to formation of anti fH responses in the human host [42]. Indeed, non-functional fHbps have demonstrated non-inferior or enhanced immunogenicity compared with wild-type proteins in transgenic mice [13], [43], although any benefit of these antigens and Ghfp will need to be assessed in clinical trials.

## Materials and Methods

### Bacterial strains and growth

The bacterial strains used in this work are shown in Table 1 and Table 2. *N. meningitidis* was grown in the presence of 5% CO_2_ at 37°C on Brain Heart Infusion (BHI, Oxoid, Basingstoke, United Kingdom) plates with 5% (vol./vol.) horse serum (Oxoid) or in BHI broth at 37°C. *N. gonorrhoeae* was grown in the presence of 5% CO_2_ at 37°C on GC agar (Oxoid) plates with Vitox (Oxoid) or in GC broth (15 g Protease peptone (Oxoid), 4 g K_2_HPO_4_, 1 g KH_2_PO_4_, 5 g NaCl per litre (Sigma Aldrich) with 10 ml Kellogg's supplement (40 g glucose, 0.5 g glutamine, 50 mg Fe(NO_3_)_9_H_2_O, 1 ml 0.2% thiamine pyrophosphate per 100 ml, Sigma Aldrich). *N. gonorrhoeae* strains were obtained from across the UK in 2012, and provided by the Sexually Transmitted Bacterial Reference Unit, Public Health England (kind gift of Dr. Ison and Dr. Quaye). *Escherichia coli* was grown on LB agar plates or LB liquid at 37°C with appropriate antibiotics.

**Table 1:** *N. meningitidis* and *N. gonorrhoeae* isolates.

*N. meningitis*					
Strain	fHbp Allele	ST	CC	Year	Group
**MC58**	1.1	74	32	1983	B
**MC58Δ** ***fhbp***					B
**H44/76**	1.1	32	32	1976	B
**H44/76Δ** ***fhbp***					B
**M1239**	3.28	437	41/44	1994	B
**m1239Δ** ***fhbp***					B
**M00.242922**	1.4	41	41/44	2000	B
**M01 240185**	1.10	4	cc11	2001	B
**M07 0240624**	1.13	6781	N/A	2007	B
**M01 240101**	1.15	1049	269	2001	B
**M07 0240839**	1.61	1163	269	2007	B
**M07 0240675**	1.292	269	269	2007	B
**M01.240013**	2.19	1159		2001	B
**M07 0240625**	2.21	1466	174	2007	Y
**M07 0240686**	2.22	7779	11	2007	C
**5/99**	2.23	1349	8	1999	B
**M07 0240877**	3.30	1214	cc269	2007	B
**M07 0240606**	3.45	213	cc213	2007	B
**M07 0240688**	3.47	1946	cc461	2007	B
**M08 0240023**	3.84	6788	cc41/44	2008	B

*N. gonorrhoeae*					
Strain	fHbp Allele	ST	CC	Year	Group
**F62**				1900	
**F62Δ** ***ghfp***					
**FA1090**				1899	

**Table 2:** *E. coli* strains used in this study.

*E. coli*				
Strain	Plasmid	Insert	Mutation	Source
**BL21**	pET28b	Ghfp		This Study
**BL21**	pET28b	Ghfp^M1^	R176Q	This Study
**BL21**	pET28b	Ghfp^M1–2^	R176Q, D199G	This Study
**BL21**	pET28b	Ghfp^M1–3^	R176Q, D199G, D212S	This Study
**BL21**	pET28b	Ghfp^M1–4^	R176Q, D199G, D212S, R288H	This Study
**BL21**	pET28b	Ghfp^M1–5^	R176Q, D199G, D212S, R288H, D318G	This Study
**BL21**	pET28b	Ghfp^M4^	R288H	This Study
**BL21**	pET28b	Ghfp ^M5^	D318G	This Study
**BL21**	pET28b	Ghfp^M4–5^	R288H, D318G	This Study
**BL21**	pET21b	fHbp V3.45		This Study
**BL21**	pET21b	fHbp^M4^	H288R	This Study
**BL21**	pET21b	fHbp^M5^	G318D	This Study
**BL21**	pET21b	fHbp^M4–5^	H288R, G318D	This Study

### Generation of mutant strains

Strain MC58Δ*fhbp* [44] and H44/76Δ*fhbp* (constructed as MC58Δ*fhbp*) were complemented with pGCC4 [45] containing *fhbp* V1.1, 1.4, 1.13, 2.16, 2.16, 3.45 and 3.47. PCR to amplify *fhbp* was performed using genomic DNA from strains listed in Table 1 and using primers in Table 3. PCR products were ligated into pGEMT (Promega) then pGCC4. Transformation of *N. meningitidis* strain MC58Δ*fHbp* was performed as described previously [46]. M1239Δ*fhbp* was constructed as MC58Δ*fhbp* and *F62Δghfp* was a kind gift of Dr. M Pizza (Novartis).

**Table 3:** Oligonucleotides used in this study.

Primer Name	Sequence (restriction site underlined)
**Ghfp F**	GCGGATCCATGACTAGGAGTAAAC
**Ghfp R**	GCGAATTCCTACTGTTTGTCGGCG
**fHbp V3.45 F**	CGCGGATCCCATATGAGCAGCGGAAGCGGAAGC
**fHbp V3.45 R**	GCCCAAGCTTCTGTTTGCCGGCGATGCC
**Ghfp M1F**	GGT GCC CTA CAG ATT GAA AAA
**Ghfp M1R**	TTT TTC AAT CTG TAG GGC AAC
**Ghfp M2F**	CTTGTCAGCGGCTTGGGCGGA
**Ghfp M2R**	TCCGCCCAAGCCGCTGACAAG
**Ghfp M3F**	CAACTGCCTGGCGGCAAAGCC
**Ghfp M3R**	GGCTTTGCCGCCAGGCAGTTG
**Ghfp M4F**	GAAGAGAAAGGCACTTACCACCTCGCCCTTTTCGGCGAC
**Ghfp M4R**	GTCGCCGAAAAGGGCGAGGTGGTAAGTGCCTTTCTCTTC
**Ghfp M5 R**	GCGAATTCCTACTGTTTGCCGGCG
**fHbp V3.45 M4F**	GAAAAAGGCACTTACCGCCTCGCTCTTTTCGGC
**fHbp V3.45 M4R**	CTTTTTCCGTGAATGGCGGAGCGAGAAAAGCCG
**fHbp V3.45 M5F**	GAAATCGGCATCGCCGACAAACAGAAGCTTGCG
**fHbp V3.45 M5R**	CTTTAGCCGTAGCGGCTGTTTGTCTTCGAACAC
**pGCC4V1.1F**	CGGTTAATTAAGGAGTAATTTTTGTGAATCGAACTGCCTTCTGCT
**pGCC4V1.4F**	CGGTTAATTAAGGAGTAATTTTTGTGAACCGAACTGCC
**pGCC4V1.15F**	CGGTTAATTAAGGAGTAATTTTTGTGAACCGAACTACC
**pGCC4V2.16F**	CGGTTAATTAAGGAGTAATTTTTGTGAACCGAACTGCCTTCTGCT
**pGCC4V3.47F**	CGG TTAATTAAGGAGTAATTTTTGTGAACCGAACTACC
**pGCC4V1.1R**	CGGTTAATTAATTATTGCTTGGCGGC
**pGCC4V1.4R**	CGGTTAATTAATTACTGCTTGGCGGCAAGAC
**PGCC4V1.15R**	CGGTTAATTAATTATTGCTTGGCGGCAAGAC
**PGCC4V2.16R**	CGGTTAATTAACTACTGTTTGCCGGCGATGC
**fHbp V1.10 F**	CGCGGATCCCATATGGTTGCCGCCGACATCG
**fHbp V1.10 R**	CCCGCTCGAGCTGCTTGGCGGCAAGAC
**fHbp V1.13 F**	CCCGCTCGAGCTGCTTGGCGGCAAGAC
**fHbp V1.13 R**	CCCGCTCGAGCTGCTTGGCGGCAAGAC
**fHbp V1.15 F**	CGCGGATCCCATATGGTCGCCGCCGACATCG
**fHbp V1.15 R**	CCCGCTCGAGTTGCTTGGCGGCAAGAC
**fHbp V1.61 F**	CGCGGATCCCATATGGTCGCCGCCGACATTG
**fHbp V1.61 R**	CCCGCTCGAGTTGCTTGGCGGCAAGAC
**fHbp V1.229 F**	CGCGGATCCCATATGGTCGCCGCCGACATCG
**fHbp V1.292 R**	CCCGCTCGAGCTGCTTGGCGGCAAGAC
**fHbp V2/V3 F**	CGCGGATCCCATATGGGCCCTGATTCTGACCGCCTGCAGCAGCGGAGGGTCGCCGCCGACATCGG
**fHbp V2 R**	CCCGCTCGAGCTGTTTGCCGGCGATGCC
**fHbp V3 R**	GCCCAAGCTTCTGTTTGCCGGCGATGCT

### Western blot analysis of fHbp

*N. meningitidis* was grown overnight and re-suspended in phosphate buffered saline (PBS). The concentration of bacteria was determined by measuring the O.D. at 260 nm of bacterial lysates in 1% SDS/0.1 M NaOH [46] and adjusted to 10^9^ CFU per ml. Samples were mixed with an equal volume of 2× SDS-PAGE loading buffer and boiled for 10 minutes, then run on SDS-PAGE gels and transferred to Immobilon PVDF membranes (Millipore). Membranes were blocked with 3% skimmed milk in 0.01% Tween in PBS (PBS-T) then incubated with primary (immune sera at a 1∶10000 dilution) and subsequently with secondary antibodies (goat anti-mouse conjugated HRP IgG, Dako, 1∶20000 dilution) all in PBS-T with 3% skimmed milk. fH binding to fHbp expressed by *N. meningitidis* or recombinant proteins was analysed by far Western blotting. Blots were incubated with normal human serum (diluted 1∶100) for 45 minutes, then incubated with anti-fH (Quidel 1∶1000 dilution), followed by rabbit anti-goat-HRP conjugated IgG (Santa Cruz 1∶20000 dilution). Binding of secondary antibodies was detected using the ECL kit (Amersham).

### Expression and purification of recombinant Ghfp and fHbp

Genes were amplified without their signal sequence by PCR with genomic DNA using primers described in Table 3. PCR products were ligated into pGEMT then into pET28a (Invitrogen, after digestion with *Bam*HΙ and *Eco*RΙ) or pET21b (Invitrogen, using *Hin*dΙΙΙ and *Xho*Ι, or *Nde*Ι and *Xho*Ι). Proteins were expressed in *E. coli* and purified using Nickel affinity chromatography followed by a HiTrapQ HP column (GE Healthcare) [13]. Mutations were introduced into *ghfp* by overlapping PCR and into *fHbp* by QuikChange Site-Directed mutagenesis (Agilent Technologies) using primers described in Table 3.

### Surface Plasmon Resonance (SPR)

SPR was performed using a Biacore 3000 (GE Healthcare). Ghfp (50 µg/ml) was first digested with 0.5 µg/ml trypsin for 2 hours at room temperature under constant shaking (300 rpm), then 0.1 mg/ml Pefabloc SC plus (Roche) was added and incubated for 10 minutes prior to dialysis against PBS. Recombinant proteins were immobilized on a CM5 sensor chip (approximately 600–1000 RU) (GE Healthcare) and increasing concentrations of fH_6–7_ (0.5 nM–32 nM) were injected over the flow channels (40 µl/min). Dissociation was allowed for 300 seconds. BIAevaluation software was used to calculate the *K*_D_.

### ELISAs

Proteins (3 µg/ml, 50 µl per well) were coated on the surface of wells (F96 maxisorp, Nunc), and after blocking with 4% BSA in PBS-T, anti-Ghfp serum was added at different dilutions and detected with goat anti-mouse HRP antibody (1∶5000 diluted) followed by substrate (Becton Dickinson). To measure fH binding to Ghfp and fHbp, proteins were coated onto wells (3 µg/ml, 50 µl per well), then incubated with fH (1 µg/ml, Sigma) and fH binding was detected using anti-fH poly clonal antibody (Quidel, 1∶1000 dilution) followed by an HRP-conjugated rabbit anti-goat IgG (Dako, 1∶5000 dilution).

### Generation of anti-Ghfp sera

Six female BALB/C mice (6–8 week old, Charles Rivers, Margate) were immunised with antigens (20 µg) absorbed to aluminium hydroxide (final concentration 3 mg/ml), 10 mM Histidine-HCL, 2 M NaCl (final concentration 9 mg/ml) in ndistilled H_2_O and mixed overnight at 4°C. The antigens were given via the intraperitioneal route on days 0, 21 and 35. Sera were collected on day 49 by terminal anaesthesia and cardiac puncture. All procedures were conducted in accordance with Home Office guidelines.

### Serum Bactericidal Assay (SBA)

*N. meningitidis* was grown on BHI plates supplemented with 1 mM IPTG overnight and suspended in PBS supplemented with 0.1% glucose (PBS-G) to a final concentration of 5×10^4^ CFU/ml. Bacteria were mixed with an equal volume of baby rabbit complement (Cedarlane) diluted 1∶10 in PBS-G. Heat inactivated serum, pooled from at least six mice was added to the wells. Control wells contained either no serum or no complement. Following incubation for 1 hour at 37°C in the presence of 5% CO_2_, 10 µl from each well was plated onto BHI plates in duplicate and the number of surviving bacteria were determined. SBA was performed with two-fold dilutions of serum starting at 1∶32. The bactericidal activity was expressed as the dilution of serum needed to kill more than 50% of bacteria in three independent experiments. Killing was calculated by comparing the number of surviving bacteria with those recovered from wells containing complement only.

### Surface protein digestion

*N. gonorrhoeae* strain F62 was grown overnight in GC liquid at 37°C then diluted 1∶20 and grown for approximately six hours until an OD A_600_ of approximately 0.5. An aliquot (1 ml) of the bacterial culture was centrifuged at 13,000× *g* then re-suspended in 300 µl of 3 ng/ml Proteinase K (Qiagen) or 3 times dilutions from this. After incubation for 30 minutes at 37°C, Pefablock SC inhibitor (Roche, final concentration 1 mM) was added for 15 minutes at room temperature. Samples were then spun and suspended in 100 µl 1× sample buffer. Digestion was assessed by Western blot analysis with antibodies against Ghfp (1∶10000 diluted), RecA (Abcam, 1∶5000 diluted), and α-Lst [18] (1∶20000) followed by goat anti-rabbit (Santa Cruz Technology, 1∶20000) or goat anti-mouse HRP conjugated IgG (Dako, 1∶20000). The relative amounts of full length protein after incubation with reducing concentrations of proteinase K was measured using AIDA software.

### Flow cytometry

Bacteria (1×10^9^) were fixed in 1 ml of 3% formaldehyde for two hours then washed with PBS. To measure fHbp expression, 5×10^7^ bacteria were incubated with 50 µl anti-Ghfp serum (diluted 1∶500) in PBS-T for 30 minutes at 4°C with shaking, washed in PBS-T then incubated with FITC conjugated goat anti-mouse antibody (DAKO, diluted 1∶50) for 30 minutes. After washing, fHbp expression was measured by flow cytometry using the FACS calibur, calculating the mean FL1 of 10000 bacteria.

## Supporting Information

Figure S1 **Alignment of Ghfp and fHbp.** Alignment of Ghfp with fHbp V1,V2 and V3. Sequence alignment is performed with the protein sequence from after the lipid modification motif in fHbp and the equivalent position in Ghfp.
(DOC)

Figure S2 **Alignment of Ghfp and fHbp V2 and V3.** Alignment of Ghfp with V2 and V3 fHbps that bind fH with *K*_D_ in the nanomolar range. The amino acids that are different in Ghpf compared to the V2 and V3 proteins are shown in red.
(DOC)
