# Supplementary material for: Distinct Binding and Immunogenic Properties of the Gonococcal Homologue of Meningococcal Factor H Binding Protein
Source: PLoS Pathog. 2013 Aug 1;9(8):e1003528. doi: 10.1371/journal.ppat.1003528 (PMC3731240; doi:10.1371/journal.ppat.1003528)
Supplement: Figure S1 — Alignment of Ghfp and fHbp. Alignment of Ghfp with fHbp V1,V2 and V3. Sequence alignment is performed with the protein sequence from after the lipid modification motif in fHbp and the equivalent position in Ghfp. (DOC) [file ppat.1003528.s001.doc]

fHbp V3.488 DKGLKSLTLEDSIPQNGTLTLSAQGAEKTFKAGDKDNSLNTGKLKNDKISRFDFVQKIEV 93

Ghfp DKGLKSLTLEASIPQNGTLTLSAQGAEKTFKAGGKDNSLNTGKLKNDKISRFDFVQKIEV 93

fHbp V2.404 DKGLQSLTLDQSVRKNEKLKLAAQGAEKTYGNG---DSLNTGKLKNDKISRFDFVQKIEV 93

fHbp V2.93 DKSLQSVTLDQSVRINENQKLVAQGAEKNYGNG---DSLNTGKLKNDKVSRLDFIRQIEV 93

fHbp V3.381 DKGLKSLTLEDSISQNGTLTLSAQGAEKTYGNG---DSLNTGKLKNDKVSRFDFIRQIEV 93

fHbp V1.120 DKGLQSLTLDQSVRKKEKLKLAAQGAEKTYGNGD---SLNTGKLKNDKVSRFDFIRQIEV 93

fHbp V1.210 DKGLKSLTLEDSISQNGTLTLSAQGAERTFKAGDKDNSLNTGKLKNDKISRFDFIRQIEV 93

**.*:*:**: *: : . .* *****:.: * ***********:**:**:::***

fHbp V3.488 DGQTITLASGEFQIYKQNHSAVVALQIEKINNPDKIDSLINQRSFLVSGLGGEHTAFNQL 153

Ghfp DGQTITLASGEFQIYKQDHSAVVALRIEKINNPDKIDSLINQRSFLVSDLGGEHTAFNQL 153

fHbp V2.404 DGQTITLASGEFQIYKQDHSAVVALQIEKINNPDKIDSLINQRSFLVSGLGGEHTAFNQL 153

fHbp V2.93 DGQLITLESGEFQIYKQDHSAVVALQIEKINNPDKIDSLINQRSFLVSGLGGEHTAFNQL 153

fHbp V3.381 DGQLITLESGEFQIYKQDHSAVVALQIEKINNPDKIDSLINQRSFLVSGLGGEHTAFNQL 153

fHbp V1.120 DGQLITLESGEFQVYKQSYSALTALQTEQEQDSEHSGKMVAKRRFRIGDIAGEHTSFDKL 153

fHbp V1.210 DGQLITLESGEFQVYKQSHSALTALQTEQVQDSEHSGKMVAKRQFRIGDIVGEHTSFGKL 153

*** *** *****:***.:**:.**: *: ::.:: ..:: :* * :..: ****:*.:*

fHbp V3.488 PDG-KAEYHGKAFSSDDAGGKLTYTIDFAAKQGHGKIEHLKTPEQNVELAAAELKADEKS 273

Ghfp PDG-KAEYHGKAFSSDDADGKLTYTIDFAAKQGHGKIEHLKTPEQNVELASAELKADEKS 273

fHbp V2.404 PSG-KAEYHGKAFSSDDAGGKLTYTIDFAAKQGHGKIEHLKTPEQNVELAAAELKADEKS 273

fHbp V2.93 PSG-KAEYHGKAFSSDDPNGRLHYSIDFTKKQGYGRIEHLKTPEQNVELASAELKADEKS 273

fHbp V3.381 PSG-KAEYHGKAFSSDDPNGRLHYSIDFTKKQGYGRIEHLKTPEQNVELASAELKADEKS 273

fHbp V1.120 PKGSSATYRGTAFGSDDAGGKLTYTIDFAAKQGYGKIEHLKSPELNVDLAAANIEQDEKH 273

fHbp V1.210 PKDVMATYRGTAFGSDDAGGKLTYTIDFAAKQGHGKIEHLKSPELNVDLAAADIKPDEKH 273

*.. * *:*.**.***..*:* *:***: ***:*:*****:** **:**:*::: ***

fHbp V3.488 HAVILGDTRYGSEEKGTYHLALFGDRAQEIAGSATVKIGEKVHEIGIAGKQ 320

Ghfp HAVILGDTRYGGEEKGTYRLALFGDRAQEIAGSATVKIGEKVHEIGIADKQ 320

fHbp V2.404 HAVILGDTRYGSEEKGTYHLALFGDRAQEIAGSATVKIGEKVHEIGIAGKQ 320

fHbp V2.93 HAVILGDTRYGGEEKGTYHLALFGDRAQEIAGSATVKIREKVHEIGIAGKQ 320

fHbp V3.381 HAVILGDTRYGGEEKGTYHLALFGDRAQEIAGSATVKIREKVHEIGIAGKQ 320

fHbp V1.120 HAVISGSVLYNQDEKGSYSLGIFGEKAQEVAGSAEVKTANGIRHIGLAAKQ 320

fHbp V1.210 HAVISGSVLYNQAEKGSYSLGIFGGQAQEVAGSAEVETANGIRHIGLAAKQ 320

**** *.. *. ***:* *.:** :***:**** *: : ::.**:* **

| **Protein variant** | **Identity to Ghfp** |
| --- | --- |
| **fHbp V1.120** | 60% |
| **fHbp V1.210** | 67% |
| **fHbp V2.93** | 81% |
| **fHbp V2.404** | 89% |
| **fHbp V3.381** | 86% |
| **fHbp V3.488** | 94% |

**Supplementary Figure 1: Alignment of Ghfp and fHbp.**

Alignment of Ghfp with the highest and lowest homologues fHbp V1,V2 and V3. Sequence alignment is performed with the protein sequence after the lipid modification motif and equivalent position of Ghfp.
