# Supplementary material for: Distinct Binding and Immunogenic Properties of the Gonococcal Homologue of Meningococcal Factor H Binding Protein
Source: PLoS Pathog. 2013 Aug 1;9(8):e1003528. doi: 10.1371/journal.ppat.1003528 (PMC3731240; doi:10.1371/journal.ppat.1003528)
Supplement: Figure S2 — Alignment of Ghfp and fHbp V2 and V3. Alignment of Ghfp with V2 and V3 fHbps that bind fH with K D in the nanomolar range. The amino acids that are different in Ghpf compared to the V2 and V3 proteins are shown in red. (DOC) [file ppat.1003528.s002.doc]

V3.13 ------MNRTAFCCLSLTTALILTACSSGGGGSGGGGVAADIGTGLADALTAPLDHKDKG 93

V3.28 -------------------------CSSGGGGSGGGGVAADIGTGLADALTAPLDHKDKG 93

Ghfp MTRSKPVNRTTFCCLSLTAGPDSDRLQQRRG--GGGGVAADIGTGLADALTAPLDHKDKG 93

V3.45 ----------------CSSGSGS----------GGGGVAADIGTGLADALTAPLDHKDKG 93

V2.22 -------------------------CSS-----GGGGVAADIGAGLADALTAPLDHKDKS 93

V2.21 -------------------------CSS-----GGGGVAADIGAGLADALTAPLDHKDKS 93

**********:***************.

V3.13 LKSLTLEDSIPQNGTLTLSAQGAEKTFKAGDKDNSLNTGKLKNDKISRFDFVQKIEVDGQ 153

V3.28 LKSLTLEDSIPQNGTLTLSAQGAEKTFKAGDKDNSLNTGKLKNDKISRFDFVQKIEVDGQ 153

Ghfp LKSLTLEASIPQNGTLTLSAQGAEKTFKAGGKDNSLNTGKLKNDKISRFDFVQKIEVDGQ 153

V3.45 LKSLTLEDSISQNGTLTLSAQGAEKTFKVGDKDNSLNTGKLKNDKISRFDFVQKIEVDGQ 153

V2.22 LQSLTLDQSVRKNEKLKLAAQGAEKTYGNGD---SLNTGKLKNDKVSRFDFIRQIEVDGQ 153

V2.21 LQSLTLDQSVRKNEKLKLAAQGAEKTYGNGD---SLNTGKLKNDKVSRFDFIRQIEVDGQ 153

*:****: *: :* .*.*:*******: *. ***********:*****:::******

V3.13 TITLASGEFQIYKQNHSAVVAL**Q**IEKINNPDKTDSLINQRSFLVS**G**LGGEHTAFNQLP**G**G 213

V3.28 TITLASGEFQIYKQNHSAVVAL**Q**IEKINNPDKTDSLINQRSFLVS**G**LGGEHTAFNQLP**G**G 213

Ghfp TITLASGEFQIYKQDHSAVVAL**R**IEKINNPDKIDSLINQRSFLVS**D**LGGEHTAFNQLP**D**G 213

V3.45 TITLASGEFQIYKQDHSAVVAL**Q**IEKINNPDKIDSLINQRSFLVS**G**LGGEHTAFNQLP**S**G 213

V2.22 LITLESGEFQIYKQDHSAVVAL**Q**IEKINNPDKIDSLINQRSFLVS**G**LGGEHTAFNQLP**S**G 213

V2.21 LITLESGEFQIYKQDHSAVVAL**Q**IEKINNPDKIDSLINQRSFLVS**G**LGGEHTAFNQLP**G**G 213

*** *********:*******:********* ************.************.*

V3.13 KAEYHGKAFSSDDPNGRLHYSIDFTKKQGYGRIEHLKTLEQNVELAAAELKADEKSHAVI 273

V3.28 KAEYHGKAFSSDDPNGRLHYSIDFTKKQGYGRIEHLKTLEQNVELAAAELKADEKSHAVI 273

Ghfp KAEYHGKAFSSDDADGKLTYTIDFAAKQGHGKIEHLKTPEQNVELASAELKADEKSHAVI 273

V3.45 KAEYHGKAFSSDDAGGKLTYTIDFAAKQGHGKIEHLKTPEQNVELASAELKADEKSHAVI 273

V2.22 KAEYHGKAFSSDDPNGRLHYSIDFTKKQGYGRIEHLKTPEQNVELASAELKADEKSHAVI 273

V2.21 KAEYHGKAFSSDDPNGRLHYSIDFTKKQGYGRIEHLKTPEQNVELASAELKADEKSHAVI 273

*************..*:* *:***: ***:*:****** *******:*************

V3.13 LGDTRYGSEEKGTY**H**LALFGDRAQEIAGSATVKIGEKVHEIGIA**G**KQ 320

V3.28 LGDTRYGSEEKGTY**H**LALFGDRAQEIAGSATVKIGEKVHEIGIA**G**KQ 320

Ghfp LGDTRYGGEEKGTY**R**LALFGDRAQEIAGSATVKIGEKVHEIGIA**D**KQ 320

V3.45 LGDTRYGSEEKGTY**H**LALFGDRAQEIAGSATVKIREKVHEIGIA**G**KQ 320

V2.22 LGDTRYGGEEKGTY**H**LALFGDRAQEIAGSATVKIREKVHEIGIA**G**KQ 320

V2.21 LGDTRYGSEEKGTY**H**LALFGDRAQEIAGSATVKIREKVHEIGIA**G**KQ 320

*******.******:******************* *********.**

**Supplementary Figure 2: Alignment of Ghfp and fHbp V2 and V3**

Alignment of Ghfp with V2 and V3 fHbps that bind fH with *K*_D_ in the nanomolar range. The amino acids that are different in Ghpf compared to the V2 and V3 proteins are shown in red.
